# Design and Biosynthesis of Ornithine 8-Containing Semaglutide Variants with a Click Chemistry-Modifiable Position 26

**DOI:** 10.1021/acssynbio.5c00132

**Published:** 2025-04-30

**Authors:** Yanli Xu, Oscar P. Kuipers

**Affiliations:** Department of Molecular Genetics, Groningen Biomolecular Sciences and Biotechnology Institute, University of Groningen, Groningen 9747 AG, The Netherlands

**Keywords:** semaglutide, ornithine, OspR, amber stop codon incorporation, RiPPs

## Abstract

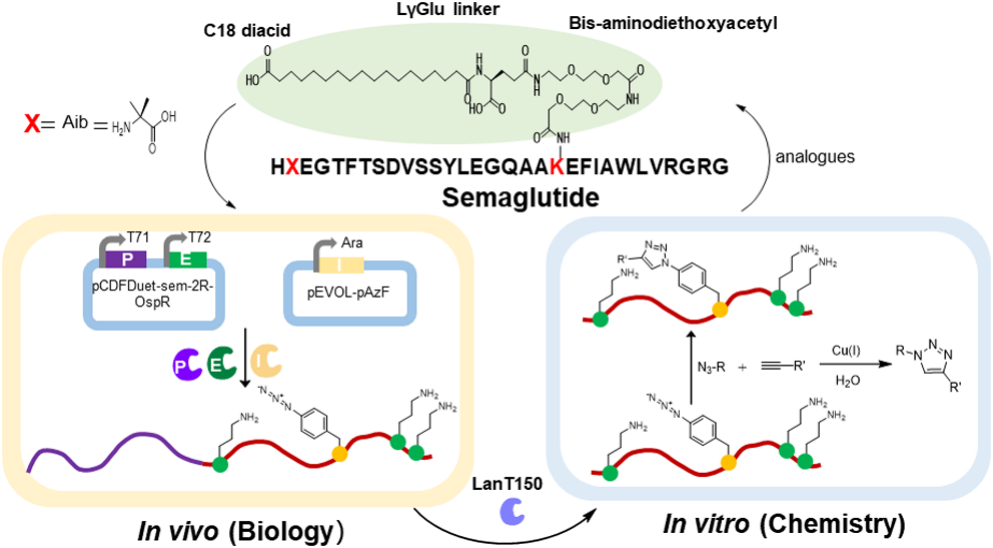

Semaglutide, a glucagon-like peptide-1 (GLP-1) receptor agonist, constitutes an effective and widely used treatment for type 2 diabetes and obesity. However, challenges such as insufficient oral bioavailability, gastrointestinal side effects, and high costs persist. Overcoming these limitations is essential for improving patient compliance and semaglutide’s safety profile. While advanced technologies such as oral delivery systems offer partial solutions, optimizing the peptide structure is crucial for addressing these issues. Establishing a rapid method to generate a large library of semaglutide mutants will enable high-throughput activity screening. In this study, we introduce a novel “Fits-In-All” approach that combines ribosomally synthesized and post-translationally modified peptide (RiPP) technology with amber stop codon incorporation to generate semaglutide variants. To counter dipeptidyl peptidase-4-mediated cleavage, our method strategically incorporates noncanonical amino acid ornithine at position 8 utilizing microbial modification enzyme OspR *in vivo*. Furthermore, functional groups are introduced by an orthogonal tRNA/aminoacyl-tRNA synthetase pair recognizing the amber stop codon at position 26, which enabled the click chemistry-based linkage of diverse groups. This approach allows for the generation of a broad array of semaglutide analogues that can be screened for optimal properties. In conclusion, this innovative approach opens new avenues for the design and synthesis of optimized peptide-based GLP-1 receptor agonists.

## Introduction

Ribosomally synthesized and post-translationally modified peptides (RiPPs) represent a diverse and expanding class of natural products known for their structural complexity and a broad range of biological activities, including antibiotics, anticancer agents, and immunosuppressants.^1−5^ Unlike nonribosomal peptides, RiPPs are initially produced as precursor peptides by the ribosome and subsequently subjected to extensive post-translational modifications (PTMs) that endow them with their final bioactive properties.^6^ These modifications, which include processes such as dehydration, hydroxylation,^7,8^ glycosylation,^9,10^ and the introduction of noncanonical amino acids,^11,12^ are typically catalyzed by dedicated enzymes encoded within the same gene cluster as the precursor peptide. Many of these enzymes have a relaxed substrate specificity, enabling their broad application in synthetic biology. Huge advancements in synthetic biology and protein engineering have thus expanded the utility of RiPP biosynthetic routes by enabling the design and production of novel peptide-based molecules with tailor-made properties.^13^ The precise *in vivo* incorporation of noncanonical amino acids, like ornithine, at specific sites offers a valuable approach to the discovery of analogues of therapeutic peptides with significantly improved pharmacodynamics and pharmacokinetics.^13,14^

**Figure 1:**
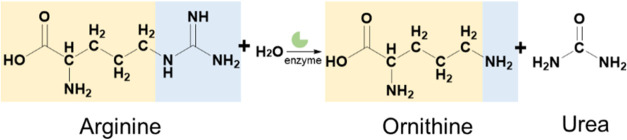
Conversion of arginine to ornithine. The structural formula of arginine is depicted on the left side of the reaction arrow, while the structural formulas of ornithine and urea are shown on the right side. The identical components of the two structures are highlighted in light yellow, while the divergent elements are highlighted in light blue. The conversion of arginine to ornithine yielded urea (42.0218 Da).

Ornithine, a noncanonical amino acid, is structurally similar to lysine (Figure 1). It plays a critical role in the design and engineering of peptides, particularly in the field of peptide therapeutics for conditions like liver disease and metabolic disorders, or for use as antibiotics.^15,16^ Incorporating ornithine into peptides can enhance their stability, resistance to enzymatic degradation, and bioactivity.^17^ This is particularly valuable in therapeutic peptides, where stability against proteolytic enzymes such as DPP-4 is crucial for maintaining bioavailability and prolonging half-life *in vivo*.^18−22^ The incorporation of such amino acids can alter peptide conformation, making it more difficult for enzymes to recognize and cleave the peptide bonds, potentially leading to improved stability.^17,23,24^ Unlike the standard 20 amino acids, ornithine is not directly genetically encoded but can be introduced into peptides through biosynthetic pathways or chemical modifications. The enzyme OspR, involved in the biosynthesis of landornamide A, catalyzes the conversion of arginine to ornithine, thus facilitating the incorporation of this noncanonical amino acid into the final peptide product.^11,12^ This specific enzymatic activity allows for the targeted modification of peptide structures, potentially enhancing their stability and bioactivity.

Incorporating the amino acid analogues via an amber stop codon incorporation offers a powerful approach for peptide modification, particularly with analogues containing functional groups that facilitate subsequent chemical modifications.^25^ This method leverages the use of an engineered tRNA and aminoacyl-tRNA synthetase pair that selectively recognizes a unique stop codon (usually the amber stop codon, UAG) as a signal to incorporate amino acid analogues. By substituting a stop codon in a target gene, analogues can be incorporated at precise locations within a peptide.^25,26^ This strategy not only expands the structural and functional diversity but also enhances the versatility of peptide engineering by enabling precise and convenient downstream functionalization.

**Figure 2:**
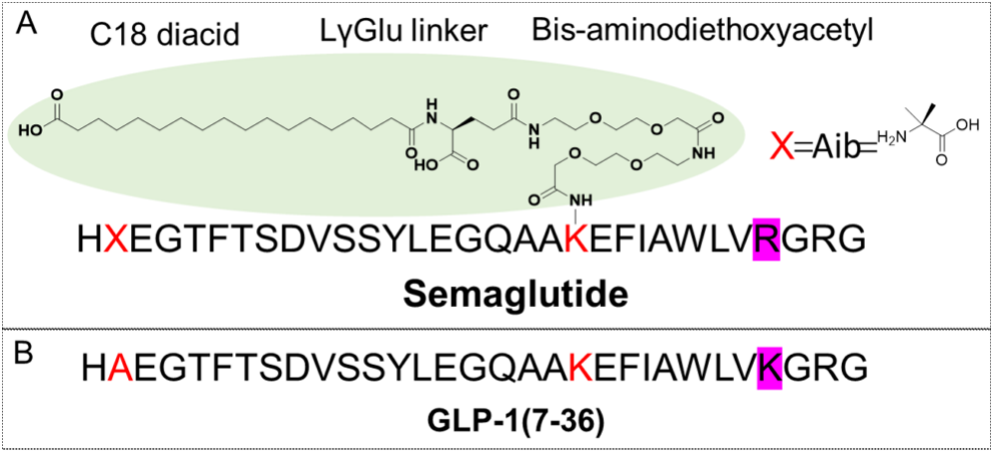
Structural schematic diagrams of wild-type GLP-1 of human^37^ and Semaglutide.^32,38^ Panel A was reused with permission from Lau et al.^32^ Copyright 2018, American Chemical Society. (A) The structures of the semaglutide exhibit two key features. First, the introduction of the noncanonical amino acid Aib (α-aminoisobutyric acid) at position 8 enhances peptide stability. This modification is represented by a red “*X*”, with the chemical structure of Aib displayed on the right side of the Figure. Second, the compound features lipidation on a lysine residue, which is marked in red. This lipid modification, aimed at improving half-life and stability, involves a fatty acid chain composed of three segments: a C18 diacid, a LγGlu linker, and bis-aminodiethoxyacetyl. The lipidated region is highlighted with a light green background. (B) Sequence of GLP-1 in humans. Apart from the nonclassical amino acid at position 8, the primary difference between semaglutide and GLP-1 lies in the lipidation at position 26. Additionally, compared to wild-type GLP-1, position 34 in semaglutide is mutated to R, highlighted with a purple background in the Figure. This modification aims to minimize the side effects of the reaction and optimize semaglutide production.

Diabetes, encompassing both Type 1 and Type 2, is a critical global health issue.^27,28^ GLP-1 (glucagon-like peptide-1) plays essential roles in glucose regulation.^29^ GLP-1 enhances insulin secretion, suppresses glucagon release, and slows gastric emptying.^30^ Semaglutide, a GLP-1 receptor agonist, is a major player in the diabetes drug market. As of recent estimates, the market size for semaglutide exceeds $10 billion.^31^ Semaglutide is a synthetic analogue of the human glucagon-like peptide-1 (GLP-1) hormone, designed to enhance its stability and prolong its biological activity.^29,32,33^ The structure of semaglutide consists of a 31-amino acid peptide backbone (Figure 2A), closely resembling native GLP-1 (Figure 2B), with specific modifications that improve its pharmacokinetic properties.^34−36^

It is especially susceptible to dipeptidyl peptidase-4 (DPP-4), which cleaves between positions 8 and 9, yielding the antagonist GLP-1 (9–36). Furthermore, GLP-1 contains 6 cleavage sites of neutral endopeptidase 24.11.^39^ A notable structural feature of semaglutide is the substitution of alanine with 2-aminoisobutyric acid at position 8, which increases resistance to dipeptidyl peptidase-4 (DPP-4) mediated degradation without providing full resistance.^18−20,22,40^ Consequently, DPP-4 inhibitors serve as a therapeutic class for the treatment of diabetes.^18^ Additionally, a large C18 fatty acid side chain is attached via a spacer to lysine at position 26, which facilitates albumin binding, thereby reducing breakdown and extending the peptide’s half-life in circulation (Figure 2A).^32,36,41−43^ These structural adaptations make semaglutide a potent and long-acting GLP-1 receptor agonist suitable for the treatment of type 2 diabetes and obesity.

Despite the use of the enhancer SNAC, semaglutide still suffers from low oral bioavailability.^38^ To address this challenge, we aimed to explore structural optimization as a potential solution. Constructing a library of semaglutide structural analogues for screening is, therefore, crucial. The introduction of nonclassical amino acids at strategic positions within the peptide, particularly at position 8, is critical for enhancing the stability and bioavailability of semaglutide.^38,41^ Various modifications of the GLP-1-derived peptide, such as glycosylation,^44−46^ lipidation,^47−49^ introduction of lysinolanine^50^ and lactam bridges,^51^ and the incorporation of d-amino acids,^52^ have been explored at different positions to enhance its therapeutic properties.^29^ However, the introduction of ornithine into therapeutic peptides has remained largely unexplored. This gap presents an opportunity for generating novel GLP-1 variants to further improve the stability and efficacy of GLP-1-based therapeutics. More importantly, the enzyme-based modification system derived from RiPPs offers high variability and flexibility, allowing for the introduction of diverse modifications at position 8, further expanding the structural diversity of semaglutide analogues. Additionally, the incorporation of clickable groups at position 26 through stop codon incorporation provides even greater structural versatility, as this position’s diversity depends on the availability of different chemical groups. Combining these two modifications would create a strong foundation for generating a broad range of semaglutide analogues, which enables efficient screening of peptide libraries with desirable properties. Furthermore, the expression of RiPPs in bacterial systems opens new possibilities for peptide delivery via the intestinal flora. This approach not only offers a potential solution for improving oral bioavailability but also presents a biosynthesis method that is simpler and more environmentally friendly than the conventional chemical synthesis currently used in the market. Here, we demonstrate the introduction of ornithine into semaglutide by the use of synthetic biology. Moreover, amino acid analogues containing azido groups were successfully incorporated at position 26 of the Orn-contained-semaglutide. This modification allows for subsequent chemical modifications, such as lipidation at this position, opening the possibility of generating a wide array of semaglutide variants for further screening.

In this study, we explored enzymatic RiPP technology to facilitate the site-specific post-translational incorporation of the noncanonical amino acid ornithine at position 8 of semaglutide *in vivo*. By coexpressing the OspR enzyme with semaglutide variants in *Escherichia coli*, we successfully achieved the conversion of arginine to ornithine at the designated site, establishing a foundation for the application of this specific RiPP biosynthetic enzyme in the modification of therapeutic peptides, offering potential benefits for their stability and therapeutic efficacy. The successful incorporation of pAzF *in vivo* at position 26 establishes a strong foundation for producing diverse semaglutide analogues via click chemistry.

## Results and Discussion

### Generation of Pre-Semaglutide by Selection of a Suitable Host

**Figure 3:**
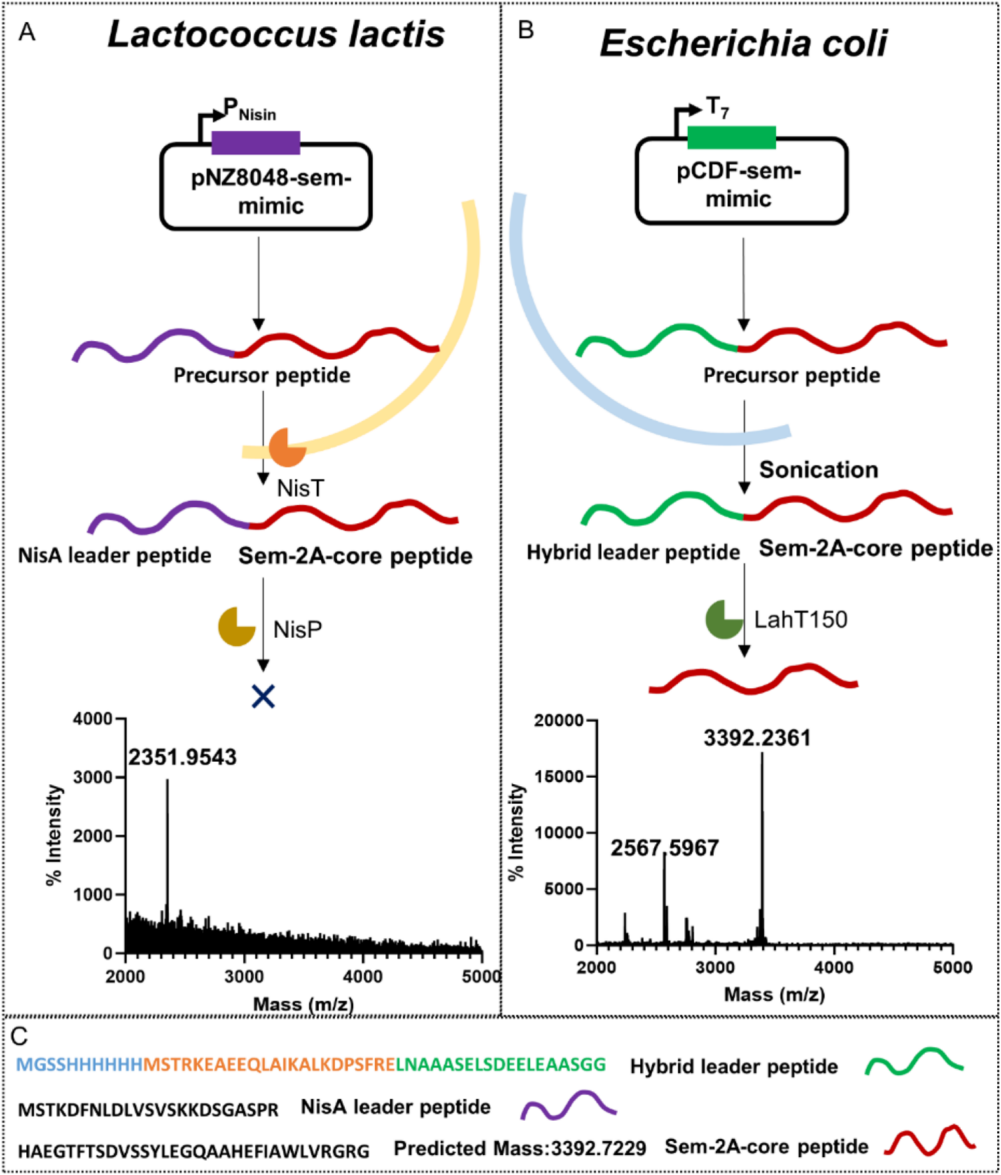
Schematic diagram of the expression of pre-semaglutide in *L. lactis* and *E. coli*. Panel A illustrates the process and MALDI-TOF mass spectrometry results for the expression and purification of Sem-2A in *L. lactis* using the NisA leader peptide. Panel B shows the corresponding process and MALDI-TOF mass spectrometry results for Sem-2A expression and purification in *E. coli* with a hybrid leader peptide. Panel C details the specific amino acid sequences of the leader and core peptides, with the NisA leader peptide highlighted in purple, the hybrid leader peptide in green, and the core peptide in red. In the hybrid leader peptide sequence, the orange region denotes the OspA leader segment, while the green region represents the ProcA3.3 leader segment. These two segments together constitute the hybrid leader peptide sequence. The blue region indicates the presence of a His tag sequence.

The host cell is crucial for the efficient expression of peptide-modifying enzymes to yield designed mature peptides. In this study, we initially selected *Lactococcus lactis* as the host, utilizing the nisin expression system. The semaglutide core-encoding sequence (*sem-2A*) was cloned downstream of the NisA leader peptide-encoding sequence and subsequently expressed in *L. lactis*. Following the leader peptide removal by NisP, the Sem-2A core peptide was analyzed by MALDI-TOF mass spectrometry (Figure 3A). However, the expected target molecular weight of the core peptide was not observed.

In the next step, we switched the expression host to *E. coli*, inserting the *sem-2A* sequence after a hybrid leader peptide-encoding sequence for expression (Figure 3C). After purification, Tricine-SDS-PAGE analysis revealed a prominent band between 5 and 10 kDa, corresponding to the precursor peptide at the expected molecular weight (Figure S1). Subsequent removal of the leader peptide by the LahT enzyme and MALDI-TOF mass spectrometry analysis yielded a distinct mass peak at 3392.24, consistent with the expected theoretical value of 3392.69 (Figure 3B and Table S2). These results demonstrate that the Sem-2A core peptide can be successfully expressed and processed in *E. coli* provided that the presence of the N-terminal hybrid leader peptide is in the precursor peptide.

### Optimization of the Production by Leader Engineering

In many RiPPs, the leader peptide not only guides the precursor peptide to the modification enzymes for precise modification of the core peptide but also keeps the peptide inactive until leader removal.

**Figure 4:**
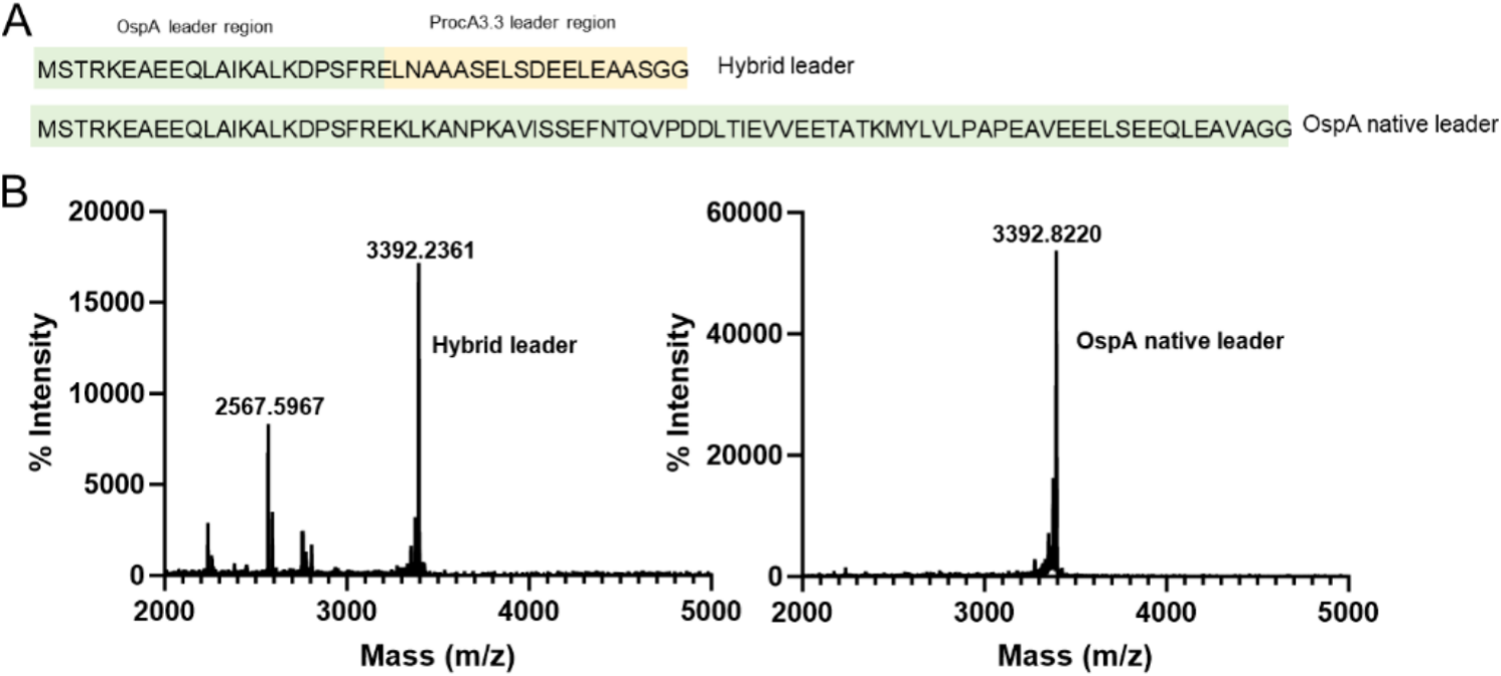
MALDI-TOF analysis of the Sem-2A core peptide expressed in *E. coli* under different leader peptides. (A) the sequences of the distinct leader peptides used. (B) The corresponding mass spectrometry results for the core peptide, with the left panel showing data with the hybrid leader peptide and the right panel with the wild-type leader peptide.

**Figure 5:**
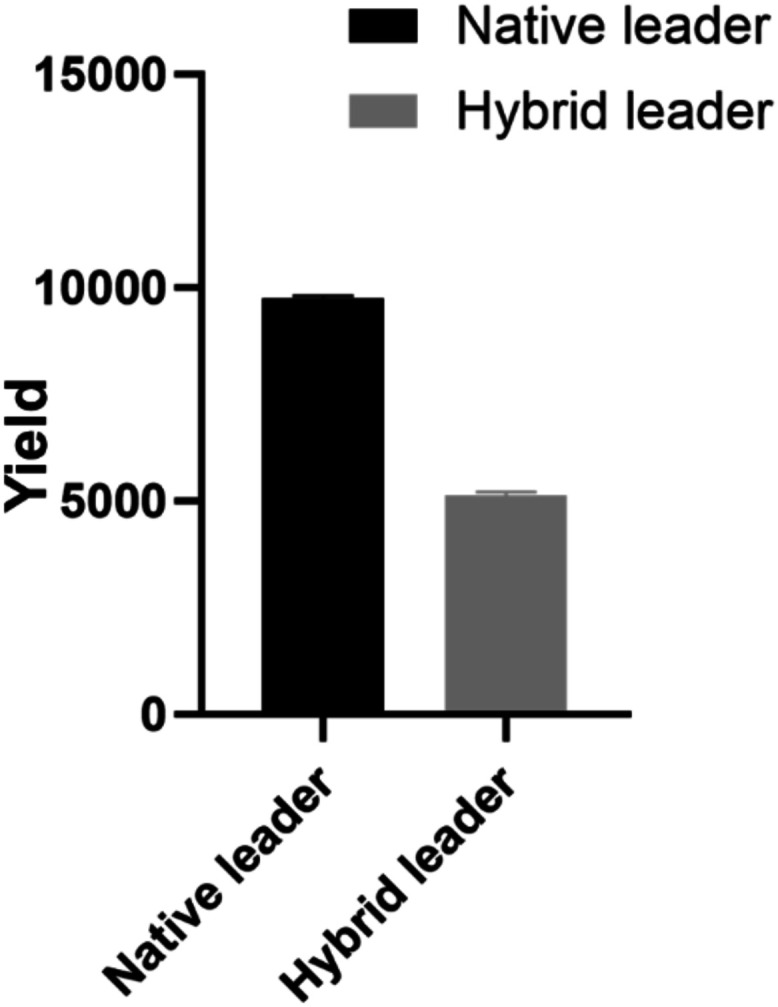
Comparison of the Sem-2A-core peptide yield with a different leader peptide. The data were collected in triplicate and quantified based on the HPLC peak area.

To investigate modifications, we introduced the sequences encoding the *Sem-2A* core peptide into pCDFDuet with the native leader peptide attached. Following expression and purification in *E. coli*, we conducted Tricine-SDS-PAGE analysis for the precursor peptides (Figure S1). The results indicate that the expression level of a precursor peptide is higher when the native leader peptide of the OspA is used compared to a hybrid leader (Figure S1A,B). After the leader peptide was removed by LahT150, MALDI-TOF mass spectrometry analysis was performed (Figure 4). The MALDI-TOF mass spectrum prominently features a peak at 3392.82, which is consistent with the expected mass of 3392.69 (Table S2). Notably, no significant additional peaks were observed, suggesting minimal degradation, whereas the hybrid leader peptide group exhibited distinct degradation fragment masses (e.g., of EGTFTSDVSSYLEGQAAHEFIAW). HPLC was then employed to quantitatively assess the core peptide yield with different leaders attached (Figure S2). The findings revealed that the yield of the core peptide behind the native leader peptide of the OspA was 1.9 times higher than that of the core peptide after the hybrid leader peptide (Figure 5).

### Site-Specific Incorporation of Ornithine Amino Acids into the Pre-Semaglutide

Given that the yield of the core peptide was higher with the native leader peptide, subsequent experiments utilized the native leader. The incorporation of noncanonical amino acids can enhance the stability of peptides. Additionally, the enzyme OspR is known to catalyze the conversion of arginine to ornithine. We chose to initially introduce ornithine into the Sem-core peptide.

**Figure 6:**
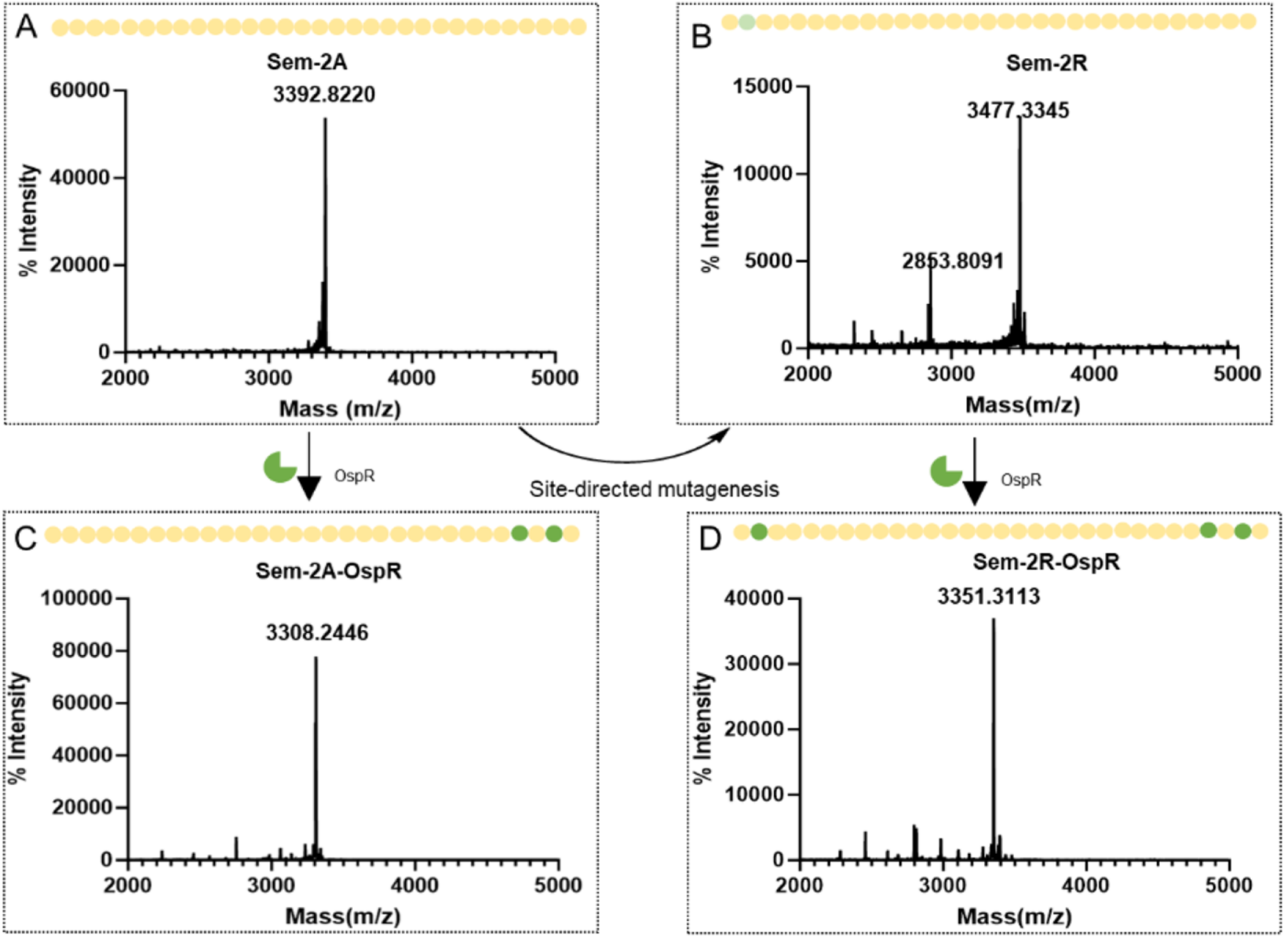
MALDI-TOF analysis of the different semaglutide mutant core peptides expressed in *E. coli*. Panel A displays the MALDI-TOF mass spectrometry results for the Sem-2A peptide, while Panel B shows the corresponding results for the Sem-2R peptide, indicating successful site-directed mutagenesis from alanine to arginine. Panels C, D present the MALDI-TOF mass spectrometry for the Sem-2A and Sem-2R, respectively, coexpressed with the OspR enzyme. In all panels, amino acids are depicted as yellow spheres, modified sites are highlighted in green, and the green partial circles represent the OspR enzyme.

To achieve this, arginine residues were introduced into the *Sem-2A* core peptide sequence through site-directed mutagenesis, yielding a *Sem-2R mutant*. Following expression and purification, the precursor peptide was analyzed using Tricine-SDS-PAGE (Figure S1). The results confirmed the successful expression of the precursor peptide of the Sem-2R mutant in *E. coli*. After the removal of the leader peptide, the purified core peptide was subjected to MALDI-TOF mass spectrometry analysis (Figure 6B). The appearance of a peak with a molecular weight of 3477.33 Da, closely matching the predicted mass of 3377.80 Da, further validated the stable expression of the Sem-2R mutant in *E. coli*.

The ability of OspR to catalyze the conversion of the Sem-2A core peptide, where two arginine residues are located at the C-terminus, was investigated. To this end, the plasmids pCDF-sem-2A and pBAD-OspR were cotransformed into *E. coli*. The expressed core peptide was subsequently purified and analyzed by MALDI-TOF mass spectrometry, as presented in Figure 6C. A new peak with a molecular weight of 3308.24 Da was detected, showing a decrease of 84.58 Da compared to that of the control group lacking OspR expression (Figure 6A). This observed mass reduction is consistent with the enzymatic conversion of two arginine residues into ornithines.

Further validation and precise identification of the conversion sites were achieved through LC-MS/MS analysis, as illustrated in Figure S3. Specifically, Figure S3A shows the LC/MS results, where the molecular weights corresponding to charge states [M+3]^3+^, [M+4]^4+^, and [M+5]^5+^ were 1103.54, 827.91, and 662.53 Da, respectively. These results confirm that the peptide was successfully expressed in *E. coli*. Moreover, the MS/MS analysis revealed that the observed molecular weights of the y^3+^, y^4+^, y^5+^, and y^6+^ fragments were consistent with the predicted values (Table S3), proving that the OspR effectively catalyzes the modification of the two C-terminal arginine residues in the Sem-2A core peptide, accurately introducing ornithine at the designated positions.

The successful expression of pCDF-Sem-2R in *E. coli*, along with the effective modification of pCDF-Sem-2A by OspR, established a solid foundation. This paved the way for the targeted introduction of an ornithine residue at position 2 of the pCDF-Sem-2R construct. Plasmids pCDF-Sem-2R and pBAD-OspR were cotransformed into *E. coli*. The resulting precursor peptide was partially purified and analyzed via Tricine-SDS-PAGE (Figure S1). The gel analysis confirmed the high-yield expression of the Sem-2R mutant precursor peptide in the presence of OspR. However, the gel could not conclusively demonstrate the conversion of arginine into ornithine. Subsequent MALDI-TOF mass spectrometry analysis (Figure 6D) revealed a peak with a molecular weight of 3351.3113 Da. This represents a reduction of 126.0235 Da compared to the control group lacking OspR expression (Figure 6B), consistent with the enzymatic conversion of three arginine residues to ornithines. In summary, MALDI-TOF mass spectrometry confirmed that all three arginine residues in the purified core peptide have successfully been converted into ornithines.

To simplify the expression system, the OspR gene was cloned into the pCDF-sem-2R mutant vector. This resulted in the creation of a new plasmid, the pCDF-sem-2R-OspR mutant. Following transformation, expression, and purification, a stable yield of the precursor peptide of pCDF-sem-2R-OspR mutant was observed (Figure S1E).

After purification of the pCDF-sem-2R-OspR mutant core peptide, LC-MS/MS analysis was conducted (Figure S4). Figure S4A displays the molecular weights corresponding to charge states [M+2]^2+^, [M+3]^3+^, [M+4]^4+^, and [M+5]^5+^, measured at 1676.82, 1118.22, 838.92, and 671.14 Da, respectively, confirming the stable expression of the precursor peptide in *E. coli*. In addition, the observed molecular weights of fragments b_2_, b_3_, b_4_, b_5_, y^3^, y^4^, y^5^, and y^6^ in the MS/MS analysis were in complete agreement with the predicted values, further validating the precise insertion and position of ornithine (Orn) as detailed in Table S4.

**Figure 7:**
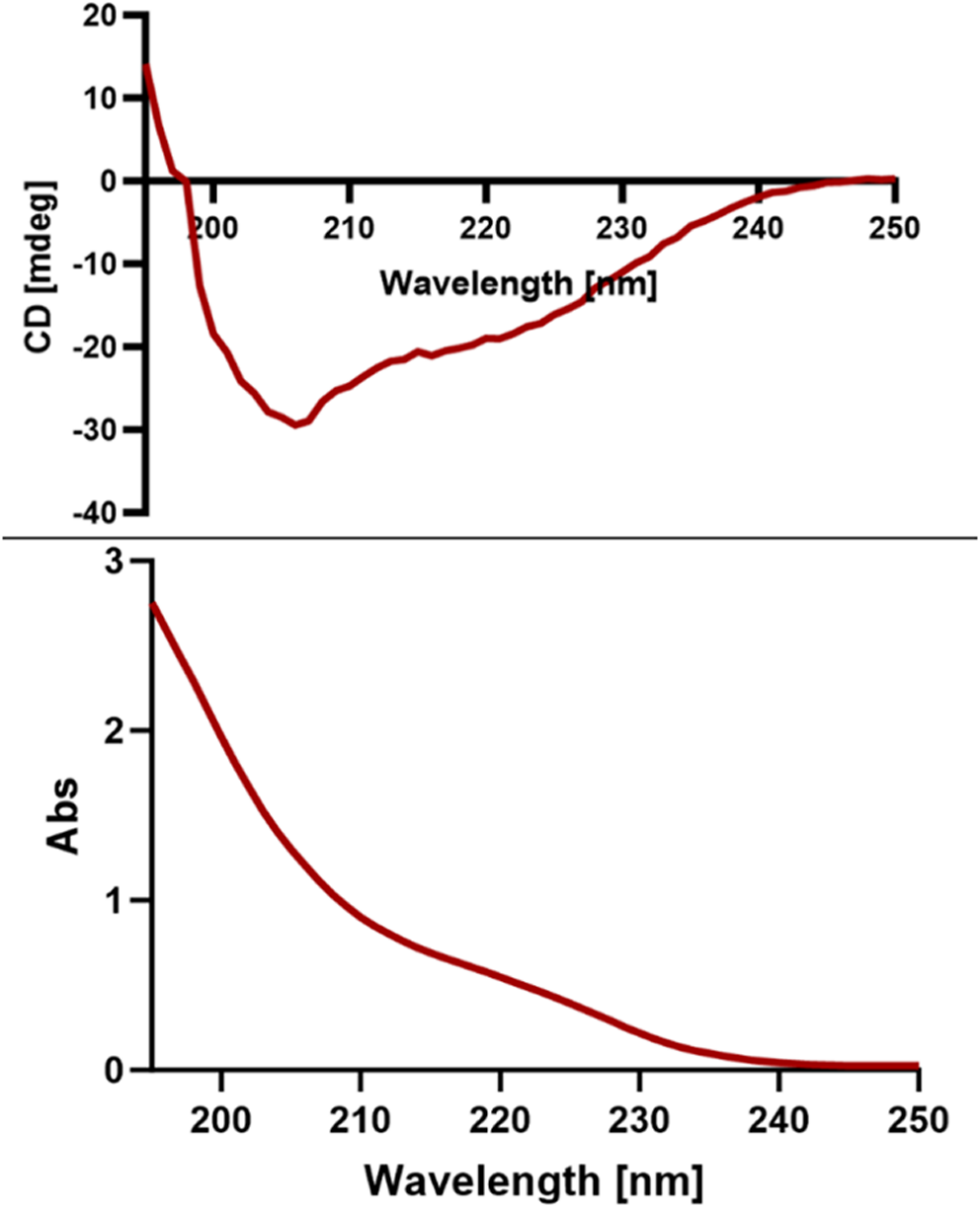
Circular dichroism (CD) spectra of the 2R-Sem-OspR peptide in MQ.

While the primary structure of a peptide is crucial for its function, its three-dimensional structure is even more important. Using LC-MS/MS, we confirmed that the OspR modification successfully incorporated Orn into the semaglutide analogue through RiPPs. However, mass spectrometry alone cannot determine whether the spatial structure of the peptide produced by RiPPs is preserved or whether the incorporation of the nonclassical amino acid Orn affects its three-dimensional conformation. Since our goal is to incorporate Orn into semaglutide, we conducted a CD spectrum analysis of the semaglutide containing Orn, as shown in Figure 7. The results indicate that the semaglutide analogue adopts an α helix structure, consistent with the known structure of GLP-1.^35^

In conclusion, comparing coexpression and single-plasmid expression, the difference in precursor peptide yield of the pCDF-sem-2R-OspR mutant was negligible. Moreover, OspR successfully modified the pCDF-sem-2R-OspR mutant construct, facilitating the targeted introduction of the noncanonical amino acid ornithine at the specific site. More importantly, the final Orn-containing semaglutide has an α-helical structure identical to that of GLP-1.

### Stability of sem-2R-OspR

The nonclassical amino acid modification at position 8 is primarily aimed at enhancing the stability of GLP-1. To evaluate the stability of semaglutide when the nonclassical amino acid Orn is substituted at position 8, compared to the wild type or Aib, we conducted a stability test on the semaglutide analogue containing Orn in human plasma over 48 h.

**Figure 8:**
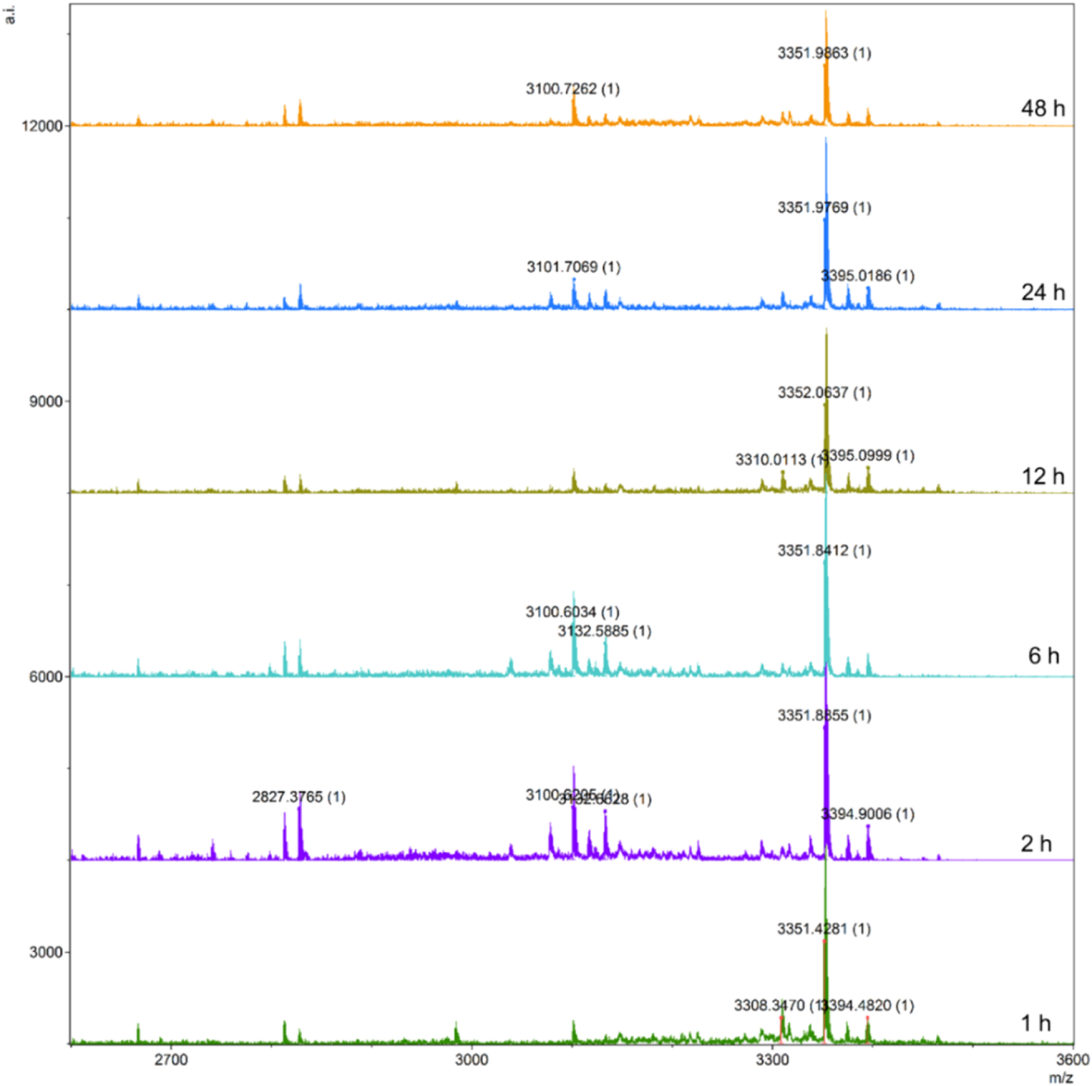
MALDI-TOF analysis of the stability of 2R-Sem-OspR in human plasma over 2 days.

The detailed procedure is outlined in the Methods section. Analysis of the results revealed that even after 48 h, a significant amount of the core peptide remained clearly detectable in the sample (Figure 8). This indicates that the incorporation of ornithine provides notable resistance to degradation, enhancing the stability of the semaglutide.

### Site-Specific Incorporation pAzF into the Orn-Contained-Semaglutide

Another essential structural feature of semaglutide is the attachment of a fatty acid side chain to a lysine residue (Figure 2, which significantly facilitates albumin binding and extends peptide *in vivo* half-lives.^41^ Given the diverse functionality enabled by click chemistry, introducing an amino acid with an azido group at this position could allow for the generation of analogues with various modifications, thus supporting subsequent high-throughput screening. Previous studies have suggested that amino acids with aromatic side chains generally displayed higher −Δ*G* values compared to aliphatic amino acids, indicating a stronger binding affinity to human serum albumin (HAS),^53^ which led us to investigate the insertion of para-azido phenylalanine (pAzF) at this position.

**Figure 9:**
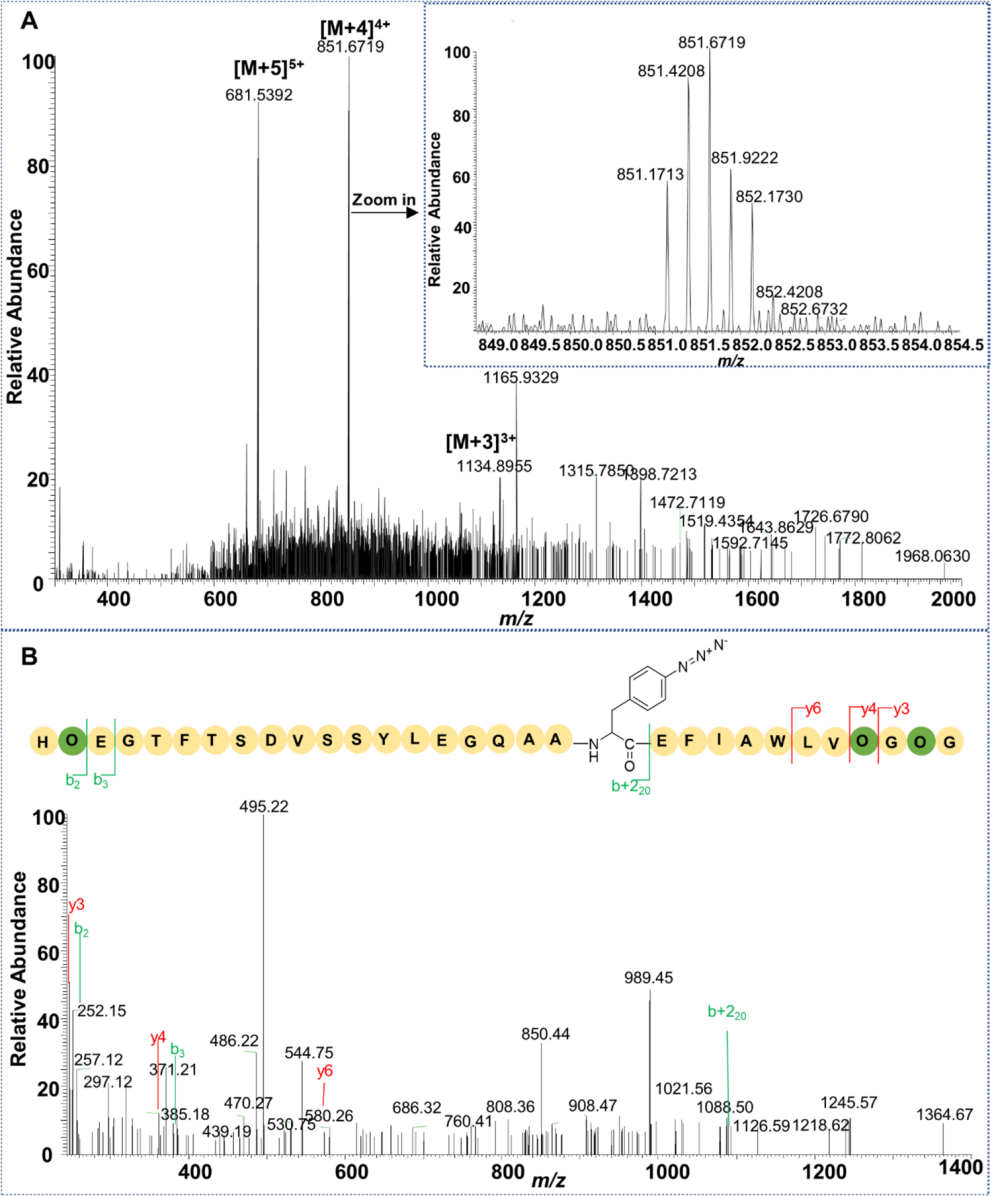
LC-MS/MS Mass spectrometry successfully demonstrates site-specific incorporation of pAzF into Sem-2R-OspR. (A) LC-MS results of the sem-2R-OspR-pAzF core peptide. The molecular weight peak for the [M+4]^4+^ charge state is zoomed in and displayed in the upper right corner. (B) LC-MS/MS results of the sem-2R-OspR-pAzF core peptide.

To achieve this, we first mutated the 26th position to an amber stop codon (UAG) via molecular cloning and then cotransformed with a plasmid containing an engineered tRNA and aminoacyl-tRNA synthetase pair.^54,55^ Tricine-SDS-PAGE analysis after expression and purification revealed that translation of precursor peptide was terminated at position 26 without the engineered tRNA and aminoacyl-tRNA synthetase pairs, while the cotransformation group continued translation and presented a band with a larger molecular weight than the control group (Figure S5). Following leader peptide removal, C18 and HPLC purification yielded the core peptide, Sem-2R-OspR-pAzF, which eluted at 50% acetonitrile. After freeze-drying, mass spectrometry was performed. Mass spectrometry data reveal peaks with molecular weights of 1134.90, 851.67, and 681.54, corresponding to the peptide’s molecular weight at [M+3]^3+^, [M+4]^4+^, and [M+5]^5+^ charge states, respectively, following the incorporation of pAzF (Figure 9A). Additionally, the MS/MS fragment analysis shows a molecular weight of 1092.53, closely matching the predicted 1092.50, further confirming the successful incorporation of pAzF into the semaglutide peptide chain containing ornithine (Figure 9B). In conclusion, these results demonstrate that pAzF can be successfully incorporated into ornithine-containing semaglutide analogues by using the stop codon incorporation method.

## Conclusions and Discussion

In conclusion, this study presents a novel approach for the site-specific enzymatic incorporation of ornithine at position 8 of the semaglutide, resulting in the first ornithine-modified semaglutide analogue. Furthermore, we successfully achieved the *in vivo* incorporation of pAzF with an azido group at position 26 in the ornithine-containing semaglutide for the first time. The combination of these two modifications provides a strong foundation for developing a diverse library of semaglutide analogues for screening specific structures or activities and paves the way for the delivery of peptides using intestinal flora. Although we successfully incorporated ornithine at position 8 and pAzF at position 26 of semaglutide within the same single peptide *in vivo*, the current methods presented in this study do not allow for precise control over the modification order, which may affect the final peptide yield. Specifically, ornithine incorporation requires a precursor peptide mutant containing arginine, while pAzF incorporation usually occurs during the initial peptide synthesis. This means that synthesizing the peptide chain with pAzF modification prior to Orn modification could theoretically enhance the yield of the final peptide. Therefore, it is worthwhile to further explore the control of the modification order through different promoters. In addition, since different vectors can influence the modification efficiency of the aaRS/tRNA_CUA_ (aminoacyl-tRNA synthetase/suppressor tRNA) pair, further investigation is needed to determine how these factors compare to the impact of modification order on overall yield.^54^ Furthermore, Exploring other natural modifications derived from RiPPs, such as β-amino acids, could further enhance stability and broaden the scope of peptide engineering.^56^

Recent research has shown that introducing lipid mimetics through *in vivo* engineering of aaRS/tRNA pairs and achieving dual lipidation of semaglutide enhances albumin binding affinity.^42,53^ Therefore, further investigation into the precise incorporation of multiple lipids at specific sites with high efficiency, as well as the simultaneous introduction of additional targeted modifications, could provide a robust foundation for developing bacterial hosts for oral peptide delivery.

## Materials and Methods

### Strains and Materials

Primers for this study were synthesized by Biolegio B.V. (Nijmegen, The Netherlands), with sequences detailed in Table S1. The Gibson Assembly Master Mix enzyme was procured from New England Biolabs. Phusion DNA Polymerase and deoxynucleotides (dNTPs) were obtained from Thermo Fisher Scientific (Waltham, MA). Amplified DNA was purified using the NucleoSpin Gel and PCR Clean-up kit (Macherey-Nagel), and plasmid DNA from newly constructed vectors was isolated using the NucleoSpin Plasmid EasyPure kit (Macherey-Nagel). All plasmid sequences were verified by Macrogen Europe (Amsterdam, The Netherlands). Unless stated otherwise, chemicals were obtained from Merck. Bacto Tryptone, Bacto Yeast Extract, and glycerol were supplied by BOOM B.V. Antibiotics were used at final concentrations of 50 μg/mL for spectinomycin (Merck) and 100 μg/mL for ampicillin (Formedium). IPTG was acquired from Thermo Fisher. *E. coli* Top10 was employed for all cloning procedures, while *E. coli* BL21(DE3) was utilized for expression experiments. Cultures were grown in LB broth (Formedium, Norfolk, U.K.) at 37 °C with 220 rpm shaking or on LB agar plates (Formedium, Norfolk, U.K.) unless otherwise specified. The *L. lactis* strain NZ9000^57^ was employed for cloning and expression. *L. lactis* cultures were grown in GM17 medium (M17 broth supplemented with 0.5% glucose) at 30 °C, with 5 μg/mL chloramphenicol or erythromycin added as needed. The pNZ plasmid, containing the NisA leader sequence,^58^ was used for the incorporation of the designed peptide sequences.

### Molecular Cloning

The genes encoding semaglutide were cloned into the pCDFDuet vector, which was modified to include either a hybrid leader or the native OspA leader sequence. Primers were used to insert the corresponding core fragments into the vector. Due to the length of these sequences, they were divided into N-terminal and C-terminal segments for incorporation, respectively. The OspR gene was integrated into the pCDFDuet-leader-Sem plasmid using the Gibson Assembly method. For *L. lactis*, the peptide sequences were introduced into the pNZ plasmid using the T4 ligation approach. All plasmid constructs were confirmed by DNA sequencing (Macrogen Europe, Amsterdam, The Netherlands). The primers utilized in this study are listed in Supporting Table S1.

### Expression of Precursor Peptides Sem-2A(R)-OspR

All constructed pCDFDuet-Sem-core plasmids were transformed into competent *E. coli* BL21(DE3) cells. The transformed cells were plated on an LB agar containing spectinomycin. Single colonies were then inoculated into 5 mL of LB broth supplemented with 50 μg/mL spectinomycin and incubated overnight at 37 °C with shaking at 220 rpm. The overnight culture was diluted 1:50 into Terrific Broth (TB: 24 g/L Bacto Yeast Extract, 12 g/L Bacto Tryptone, 5 mL/L glycerol, 0.017 M KH_2_PO_4_, 0.072 M K_2_HPO_4_) supplemented with a 50 μg/mL for spectinomycin. The cultures were grown at 37 °C and 220 rpm until an OD_600_ between 1.0 and 2.0. After cooling on ice, peptide expression was induced with 1 mM IPTG (final concentration), and the cultures were incubated at 18 °C for approximately 20 h at 200 rpm. For coexpression of Sem-2R and OspR, induction was carried out with 1 mM arabinose and 1 mM IPTG, while all other conditions remained consistent with the peptide expression protocol described above.

To express semaglutide mimics in *L. lactis*, the pNZ-NisA leader-Sem system was utilized. *L. lactis* NZ9000, carrying the *nisT* plasmid, was electroporated with pNZ-NisA leader-Sem and cultured overnight on GM17 agar plates containing 5 μg/mL chloramphenicol and 5 μg/mL erythromycin at 30 °C. A single colony was selected and grown in 4 mL of GM17 medium until the culture reached an OD_600_ of approximately 0.4, at which point peptide expression was induced by adding 5 ng/mL nisin, and the cultures were incubated at 30 °C overnight.

### Coexpression of Precursor with Amber Suppressor tRNA/aaRS

The pCDFDuet-26stop-2R-sem-OspR constructs, containing 26 position amber stop codon mutants in the core sequence, were cotransformed with the pEVOL-pAzF plasmid (incorporates Phe analogues in response to the amber stop codon, in this study pAzF was used) in *E. coli* BL21(DE3) cells. The pCDFDuet-26stop-2R-sem-OspR wild-type sequence was independently transformed into BL21(DE3) cells as a control group. Single colonies were picked for expression in TB media containing spectinomycin (50 μg mL^–1^) and chloramphenicol (25 μg mL^–1^) and grown overnight. Cultures (250 mL) were grown to OD_600_ = 0.8–1.0 and induced with IPTG (1 mM) and arabinose (0.02%) and supplemented with pAzF (1 mM). The cells were cultured for 20 h with shaking of 200 rpm at 18 °C. After 20 h, cultures were pelleted, and purification was performed.

### Peptide Purification

The cells (from 100 mL culture) were harvested by centrifugation (4 °C, 10,000*g*, 5 min), resuspended in 20 mL of lysis buffer (20 mM NaH_2_PO4, 300 mM NaCl, 10 mM imidazole, pH 7.4), and lysed by sonication (10 s ON, 10 s OFF, 45–55% amplitude, 10–15 min). The lysate was obtained by centrifugation (4 °C, 10,000 rpm, 30 min) and filtered through 0.45 μM filters. The sample was loaded on an equilibrated Ni-NTA agarose column and mixed well. The resin was washed with 10 CV wash buffer (20 mM NaH_2_PO_4_, 300 mM NaCl, 40 mM imidazole, pH 7.4) and eluted with 5 mL of elution buffer (20 mM NaH_2_PO_4_, 300 mM NaCl, 500 mM imidazole, pH 7.4). The sample was desalted through an equilibrated PD-10 desalting column with Sephadex G-25 resin (GE Healthcare) and eluted in 7 mL of 50 mM Tris-HCl (pH 8.0). The core peptide was released from the His_6_-tagged leader by 1:20 addition of LahT150 protease (containing 1 mM DTT) for 2 h at 37 °C. Core peptide mixtures were centrifuged (4 °C, 10,000 rpm, 15 min), filtered through 0.45 μM filters, and further purified with a second equilibrated Ni-NTA agarose column. The sample was loaded and mixed well, and the flow-through was collected directly, followed by the addition of 7 mL of second His_6_-tag buffer (20 mM Tris, 300 mM NaCl, pH 7.5). Core peptides were further purified by an open column with C18 resin (Waters), washed with 3 mL 0.1% trifluoroacetic acid (TFA) in acetonitrile (ACN), and equilibrated with 5 mL Milli-Q + 0.1% TFA. After sample loading, the column was washed with 10 mL 15% ACN + 0.1% TFA, and the core peptide was eluted with 8 mL 60% ACN + 0.1% TFA and lyophilized. Lyophilized core peptides will serve for further analysis.

To purify semaglutide mimics in *L. lactis*, the supernatant was collected after overnight incubation by centrifugation at 10,000*g* for 15 min. The precursor peptide was precipitated using 10% TCA on ice for 2 h. The resulting precipitate was centrifuged at 10,000*g* for 40 min at 4 °C, washed with 10 mL of ice-cold acetone to remove residual TCA, and then either air-dried and resuspended in 0.2 mL of 0.05% aqueous acetic acid for subsequent analysis. For the pellet, it was resuspended in 10 mL of lysis buffer containing 20 mg/mL lysozyme and lysed by sonication for 30 min. Ni-NTA purification was performed, and the buffer was exchanged with 50 mM Tris-HCl, pH 6.0, using a PD-10 desalting column with Sephadex G-25 resin (GE Healthcare). The leader peptide was then removed by adding NisP *in vitro*. Core peptides were further purified using an open column with C18 resin, washed with 5 mL of 0.1% trifluoroacetic acid (TFA) in acetonitrile (ACN), and equilibrated with 10 mL of Milli-Q water containing 0.1% TFA. After the sample was loaded, the column was washed with 10 mL of 15% ACN + 0.1% TFA, and the core peptide was eluted with 5 mL of 60% ACN + 0.1% TFA, followed by lyophilization. The lyophilized core peptides were used for further analysis. Both the lyophilized sample and the sample obtained from the supernatant from TCA precipitation were analyzed by MALDI-TOF mass spectrometry.

The lyophilized sample was reconstituted in Milli-Q (MQ) water. Following reconstitution, the sample was filtered through a 0.2 μm membrane to remove particulate matter. Peptide purification was performed using an Agilent 1260 Infinity high-performance liquid chromatography (HPLC) system equipped with a Phenomenex Aeris C18 column (250 mm × 4.6 mm, 3.6 μm particle size, and 100 Å pore size). The mobile phase consisted of acetonitrile (MeCN) and MQ water, with a linear gradient of 45–65% MeCN over a 20 min period at a flow rate of 1 mL/min. Peptides, including semaglutide-related mutants, eluted between 50 and 53% MeCN.

### MALDI-TOF Mass Spectrometry

For MALDI-TOF analysis, 1 μL of the sample was applied to the MALDI target plate and allowed to dry. Following this, 1 μL of matrix solution (comprising 5 mg/mL α-cyano-4-hydroxycinnamic acid in 50% acetonitrile with 0.1% trifluoroacetic acid) was layered on top of the dried sample. Matrix-assisted laser desorption/ionization time-of-flight (MALDI-TOF) mass spectrometry was then conducted using a 4800 Plus MALDI-TOF/TOF Analyzer (Applied Biosystems) in reflector positive mode.

### LC-MS/MS Spectrometry

LC-MS/MS analyses were performed with a Shimadzu LC20 XR-series HPLC system with binary LC20ADXR pumps interfaced to a Q Exactive Plus hybrid quadrupole-orbitrap mass spectrometer (Thermo Scientific). Agilent Pursuit XRs 3 C8 column with 2.6 μm 100 Å particles (Phenomenex) was used for separation. The column and autosampler temperatures were set at 50 and 10 °C, respectively. The injection volume was 5 μL, and the flow was set at 0.3 mL/min. The mobile phase A was MQ with 0.1% formic acid and mobile phase B was acetonitrile with 0.1% formic acid. A linear gradient was used: 0–1 min 2% B, 2–5 min linear increase to 45% B, 5–7 min linear increase to 95% B, 23.7–8 min held at 95% B, 27–27.1 min decrease to 0.5% B, and 8.1–9.5 min held at 0.5% B. MS and MS/MS analyses were performed with electrospray ionization in positive mode at a spray voltage of 3.5 kV, and sheath and an auxiliary gas flow were set at 60 and 11, respectively. The ion transfer tube temperature was 320 °C. Spectra were acquired in data-dependent mode with a survey scan at *m*/*z* 300–2000 at a resolution of 70 000, followed by MS/MS fragmentation of the top 5 precursor ions at a resolution of 17500. A normalized collision energy (NCE) of 30 was used for fragmentation, and fragmented precursor ions were dynamically excluded for 10 s. LC-MS/MS data was analyzed by the Xcalibur software.

### Circular Dichroism (CD) Spectroscopy Assay

The Sem-2R-OspR was dissolved in MQ to a roughly final concentration of 100 μM. After putting the sample in a quartz cuvette with a 1 mm path length, CD spectra were recorded using a Jasco J-815 spectropolarimeter at room temperature. The spectra were collected over the wavelength range of 195–250 nm with a bandwidth of 1 nm and a scanning speed of 100 nm/min with three accumulations. The raw CD data were baseline-corrected using an MQ as a reference. The collected data was analyzed by GraphPad Prism 10.

### Stability Assay in Human Plasma

Human plasma was obtained from Innovative Research Company and stored at −80 °C until use. Before the experiment, the plasma was thawed at 37 °C for further use. Sem-2R-OspR were prepared in MQ and diluted to a final concentration of 100 μM in human plasma. The samples were incubated at 37 °C in a water bath. Aliquots of the peptide-plasma mixture were collected at 1, 2, 6, 12, 24, and 48 h. At each time point, 50 μL of the sample was withdrawn and immediately mixed with 150 μL of 100% acetonitrile. The samples were then centrifuged at 12,000*g* for 15 min at 4 °C. The filtered supernatant (0.2 μm membrane) was purified using a ZipTip with a 50 μL sample volume, and the purified sample was then analyzed by MALDI-TOF. Each time point experiment was performed in triplicate.

## Supporting Information Available

Overview of primers used; overview of mass summary of Sem-2R-OspR; Tricine-SDS-PAGE for Sem-related precursor peptides; HPLC spectrum of Sem-related core peptides during purification and quantification; and LC-MS/MS spectrometry data (PDF)
