# Supplementary material for: Design and Biosynthesis of Ornithine 8-Containing Semaglutide Variants with a Click Chemistry-Modifiable Position 26
Source: ACS Synth Biol. 2025 Apr 30;14(5):1790–801. doi: 10.1021/acssynbio.5c00132 (PMC12090216; doi:10.1021/acssynbio.5c00132)
Supplement: Supplementary file 1 — sb5c00132_si_001.pdf [file sb5c00132_si_001.pdf]

Supporting Information

Design and Biosynthesis of Ornithine 8-containing Semaglutide Variants with a Click Chemistry-Modifiable Position 26

Yanli Xu[1], Oscar P. Kuipers[1*]

[1]Department of Molecular Genetics, Groningen Biomolecular Sciences and Biotechnology Institute, University of Groningen, Nijenborg 7, Groningen, 9747 AG, The Netherlands.

* Correspondence: o.p.kuipers@rug.nl (Oscar P. Kuipers)

Table S1 Summary of Primers used in this study

Primer	Sequences (5' to 3')	Purpose
sem-1-hybrid-Fw	CACCTCTGACGTTTCTTCTTACCTGGAAGGTTAAGCTTGCGGCCGCATAATGCTTAAGTCG	pCDF-hybrid-sem-2A-construct-N terminal
sem-1-hybrid-Rv	GGTAAGAAGAAACGTCAGAGGTGAAGGTACCTTCAGCGTGGCCACCGCTGGCTGCTTCCAGTTCTTC	pCDF-hybrid-sem-2A-construct-N terminal
sem-2-hybrid-Fw	TTCATCGCTTGGCTGGTTCGTGGTCGTGGTTAAGCTTGCGGCCGCATAATGCTTAAGTC	pCDF-hybrid-sem-2A-construct-C terminal
sem-2-hybrid-Rv	ACGAACCAGCCAAGCGATGAATTCGTGAGCAGCCTGACCTTCCAGGTAAGAAGAAACGTCAGAGG	pCDF-hybrid-sem-2A-construct-C terminal
sem-1-native-Fw	CTTCACCTCTGACGTTTCTTCTTACCTGGAAGGTTAAAAGCTTGCGGCCGCATAATGCTTAAG	pCDF-native-sem-2A-construct-N terminal
sem-1-native-Rv	GAAGAAACGTCAGAGGTGAAGGTACCTTCAGCGTGGCCACCAGCGACAGCTTCTAATTGTTC	pCDF-native-sem-2A-construct-N terminal
sem-2-native-Fw	TTCATCGCTTGGCTGGTTCGTGGTCGTGGTTAAAAGCTTGCGGCCGCATAATGCTTAAG	pCDF-native-sem-2A-construct-C terminal
sem-2-native-Rv	ACGAACCAGCCAAGCGATGAATTCGTGAGCAGCCTGACCTTCCAGGTAAGAAGAAACGTCAGAGG	pCDF-native-sem-2A-construct-C terminal
sem-1-lactics-Fw-P	CTGACGTTTCTTCTTACCTGGAAGGTTAAGCTTTCTTTGAACCAAAATTAGAAAACC	pNZ-sem-2A-construct-N terminal
sem-1-lactics-Rv	AGGTGAAGGTACCTTCAGCGTGGCGTGGTGATGCACCTGAATCTTT	pNZ-sem-2A-construct-N terminal
sem-2-lactics-Fw-P	CTTGGCTGGTTCGTGGTCGTGGTTAAGCTTTCTTTGAACCAAAATTAGAAAACC	pNZ-sem-2A-construct-C terminal
sem-2-lactics-Rv	CGATGAATTCGTGAGCAGCCTGACCTTCCAGGTAAGAAGAAACGTCAGA	pNZ-sem-2A-construct-C terminal
2R-sem-mutant-1-Fw	GGTACCTTCACCTCTGACGTTTCTTCTTACCTGGAAGGTCAGGCTGCTCAC	pCDF-native-sem-2R-construct-N terminal
2R-sem-mutant-1-Rv	GAAACGTCAGAGGTGAAGGTACCTTCACGGTGGCCACCGCTGGCTGCTTC	pCDF-native-sem-2R-construct-N terminal
2R-26stop-Fw	CTGCTTAGGAATTCATCGCTTGGCTGGTTC	pCDF-native-sem-2R-26-stop-construct-N terminal
2R-26stop-Rv	GATGAATTCCTAAGCAGCCTGACCTTCCAGGTAAG	pCDF-native-sem-2R-26-stop-construct-N terminal
OspR -Fw	ATGGCTAAGATTCCGTTTTATATCATGG	pCDF-native-sem-2R-OspR-construct
OspR -Rv	TTAGACCTCTACTAAAACTTTTTTAGACAG	pCDF-native-sem-2R-OspR-construct
pCDF-T72-OspR -Fw	AAGTTTTAGTAGAGGTCTAAGCAGATCTCAATTGGATATC GGCCG	pCDF-native-sem-2R-OspR-construct
pCDF-T72-OspR -Rv	TAAAACGGAATCTTAGCCATATGTATATCTCCTTCTTATA CTTAACTAATATACTAAGATGGGG	pCDF-native-sem-2R-OspR-construct

Table S2 Summary of predicted mass of Semaglutide mutants

Name	Sequences of Core	Mass	1R to Orn	2R to Orn	3R To Orn
Sem-2A	HAEGTFTSDVSSYLEGQAAHEFIAWLVRGRG	3392.69	-	-	-
Sem-2R	HREGTFTSDVSSYLEGQAAHEFIAWLVRGRG	3477.80	-	-	-
Sem-2A-OspR	HAEGTFTSDVSSYLEGQAAHEFIAWLVRGRG	3392.69	3350.69	3308.69	-
Sem-2R-OspR	HREGTFTSDVSSYLEGQAAHEFIAWLVRGRG	3435.80	3393.80	3351.80	3309.80

Note: The masses are calculated by Expasy (https://web.expasy.org/compute_pi/)

Table S3 LC-MS/MS results of Sem-2A-OspR

Species	Predicted mass (Da)	Observed mass (Da)
b3	338.15	338.15
b4	395.17	395.16
b5	496.22	496.22
b6	643.28	643.29
b7	744.33	744.33
b8	831.36	831.37
b9	946.39	946.40
b10	1045.46	1045.45
b11	1132.49	1132.51
b12	1219.52	1219.52
b13	1382.59	1382.61
b14	1495.67	1495.69
b15	1624.71	1624.72
b16	1681.73	1681.77
b17	1809.79	1809.78
b18	1880.83	1880.80
b19	1951.87	1951.85
y16	1683.90	1683.86
y15	1626.88	1626.71
y12	1356.75	1356.70
y11	1219.69	1219.52
y10	1090.64	1090.48
y6	573.38	573.38

y5	460.29	460.29
y4	361.22	361.22
y3	247.14	247.14

Note: The masses are calculated by ProteinProspector.

https://prospector.ucsf.edu/prospector/mshome.htm

Table S4 LC-MS/MS results of Sem-2R-OspR

Species	Predicted mass (Da)	Observed mass (Da)
b2	252.15	252.15
b3	381.19	381.19
b4	438.21	438.21
b5	539.26	539.26
b6	686.33	686.32
b7	787.38	787.38
b8	874.41	874.40
b9	989.43	989.44
b10	1088.50	1088.48
b11	1175.53	1175.51
b12	1262.57	1262.58
b13	1425.63	1425.64
y8	830.49	830.49
y7	759.46	759.46
y6	573.38	573.38
y5	460.29	460.29
y4	361.22	361.22
y3	247.14	247.14
y2	190.12	190.12
y1	76.04	76.02

Table S5 LC-MS/MS results of Sem-2R-OspR-pAzF

Species	Predicted mass (Da)-M	Predicted mass (Da)-(2)R to Orn(+)	Observed mass (Da)	Predicted mass (Da)-(2)R to Orn(+)-b+2/y+2	Observed mass (Da)
b2	294.17	252.15	252.15		
b3	423.21	381.19	381.18		
b4	480.23	438.21	439.19		
b5	581.28	539.26	-		
b6	728.35	686.33	686.32		
b7	829.40	787.38	-		
b8	916.43	874.41	-		
b9	1031.45	989.43	989.45		
b10	1130.52	1088.50	1088.50		
b11	1217.55	1175.53	-		
b12	1304.59	1262.57	-		
b13	1467.65	1425.63	-		
b19	2036.93	1994.93			
b20	2184.00	2142.00	-	1071.50/1092.50	1092.53
y17	1896.98	1812.94	-		
y16	1767.94	1683.90	-		
y15	1710.92	1626.88	-	813.94/834.94	812.94/834.39
y14	1582.86	1498.82	-	749.91/770.91	
y13	1511.82	1427.78	-	714.39/735.39	
y12	1440.79	1356.75	-	678.88/699.88	-
y11	1303.73	1219.69	1218.62	610.34	-
y10	1174.68	1090.64	1089.52	545.82	545.25

y9	1027.62	943.58	943.39	472.29	-
y8	914.53	830.49	830.47	415.75	-
y7	843.50	759.46	760.41		
y6	657.42	573.38	573.38		
y5	544.33	460.29	-		
y4	445.26	361.22	361.22		
y3	289.16	247.14	247.14		
y2	232.14	190.12	-		
y1	76.04	76.04	-		

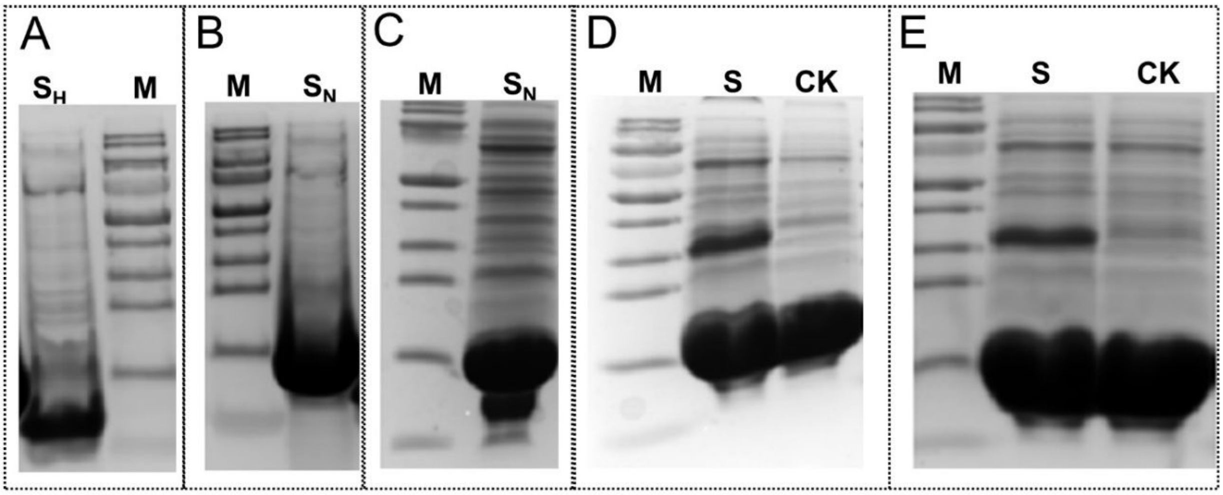

Figure S1 The yields of Sem-2A and Sem-2R precursor peptides with different leaders and co-expression with OspR in *E. coli* by tricine gels

Box A: Depicts the yield of the Sem-2A precursor peptide in E. coli when fused to a hybrid leader peptide. "S_H" indicates the mature Sem-2A peptide with the hybrid peptide attached. **Box B**: Illustrates the yield of the Sem-2A precursor peptide in *E. coli* when fused to its native leader peptide. "S_N" denotes the mature Sem-2A peptide with the native leader peptide attached. **Box C**: Shows the yield of the Sem-2R precursor peptide in E. coli with the native leader peptide attached. **Box D**: Represents the yield of the Sem-2R precursor peptide in *E. coli*, co-transformed with the OspR expression plasmid while retaining the native leader peptide. "S" corresponds to the co-transformation with plasmids pCDF-Sem-2R and pBAD-OspR, whereas "CK" indicates co-transformation with plasmids pCDF-Sem-2R and the empty vector pBAD. **Box E**: Depicts the yield of the Sem-2R precursor peptide in E. coli with the native leader peptide attached and transformed with the OspR expression plasmid. "S" refers to the plasmid pCDF-

Sem-2R-OspR, and "CK" indicates the plasmid pCDF-Sem-2R (without OspR). "M" denotes the molecular weight marker (ladder).

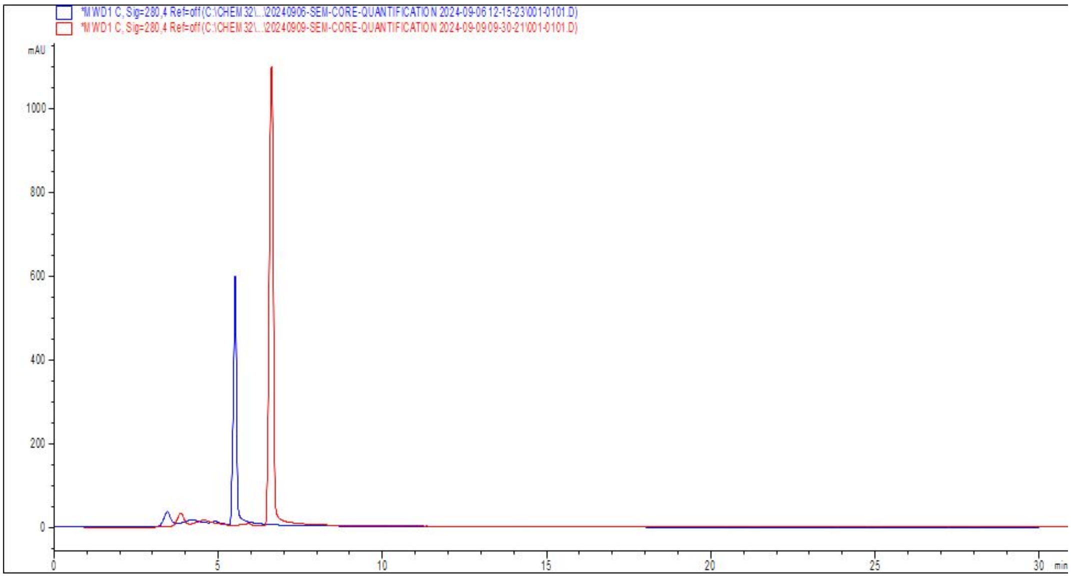

Figure S2 HPLC chromatograms of purified sem-2A core peptide with different leader attached are shown.

The blue line represents the hybrid leader peptide while the red line represents the OspA native leader peptide. Each experiment was conducted in triplicate, with quantification based on peak area measurements.

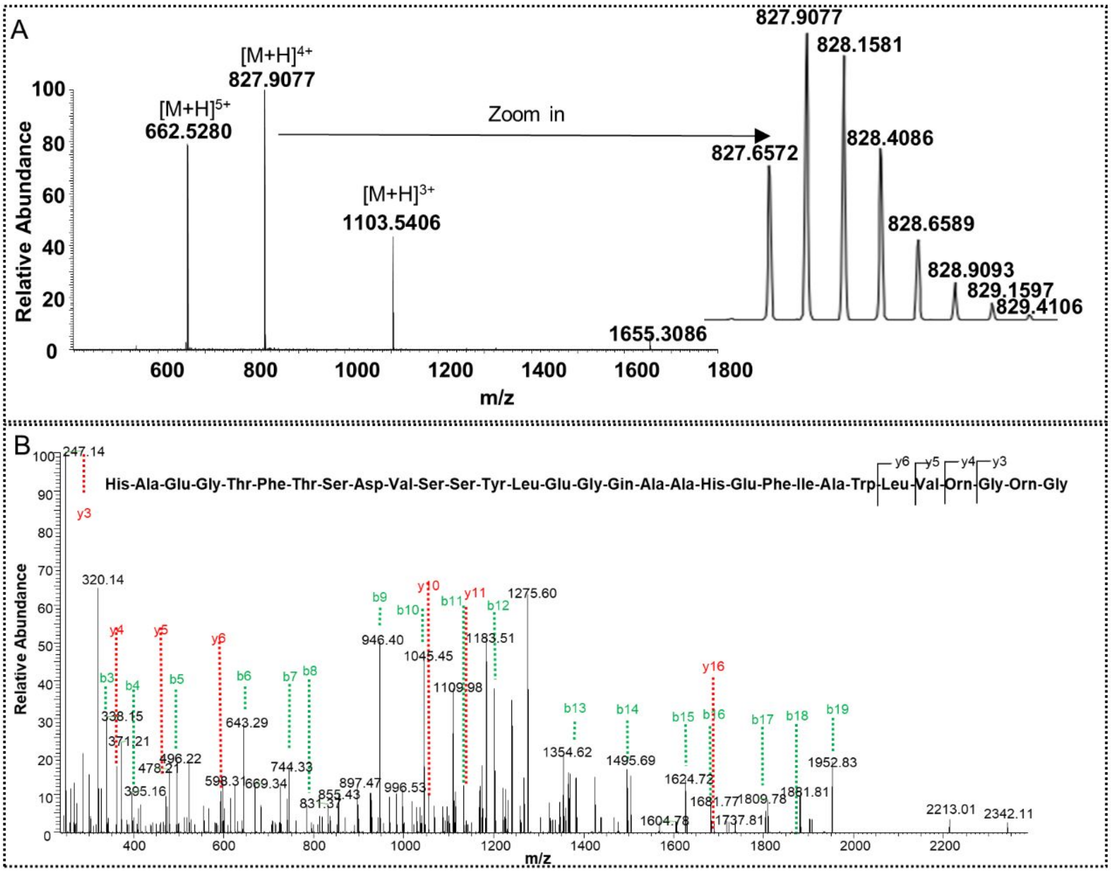

Figure S3. Mass spectrometry of core peptide Sem-2A with OspR.

(A) LC-MS result of core peptide Sem-2A with OspR. (B) LC-MS/MS result of core peptide Sem-2A with OspR. Figure S3A shows the LC/MS results, where the molecular weights corresponding to charge states 3+, 4+, and 5+ were 1103.54 Da, 827.91 Da, and 662.53 Da, respectively. The MS/MS analysis revealed that the observed molecular weights of the y3+, y4+, y5+, and y6+ fragments were consistent with the predicted values (Table S3).

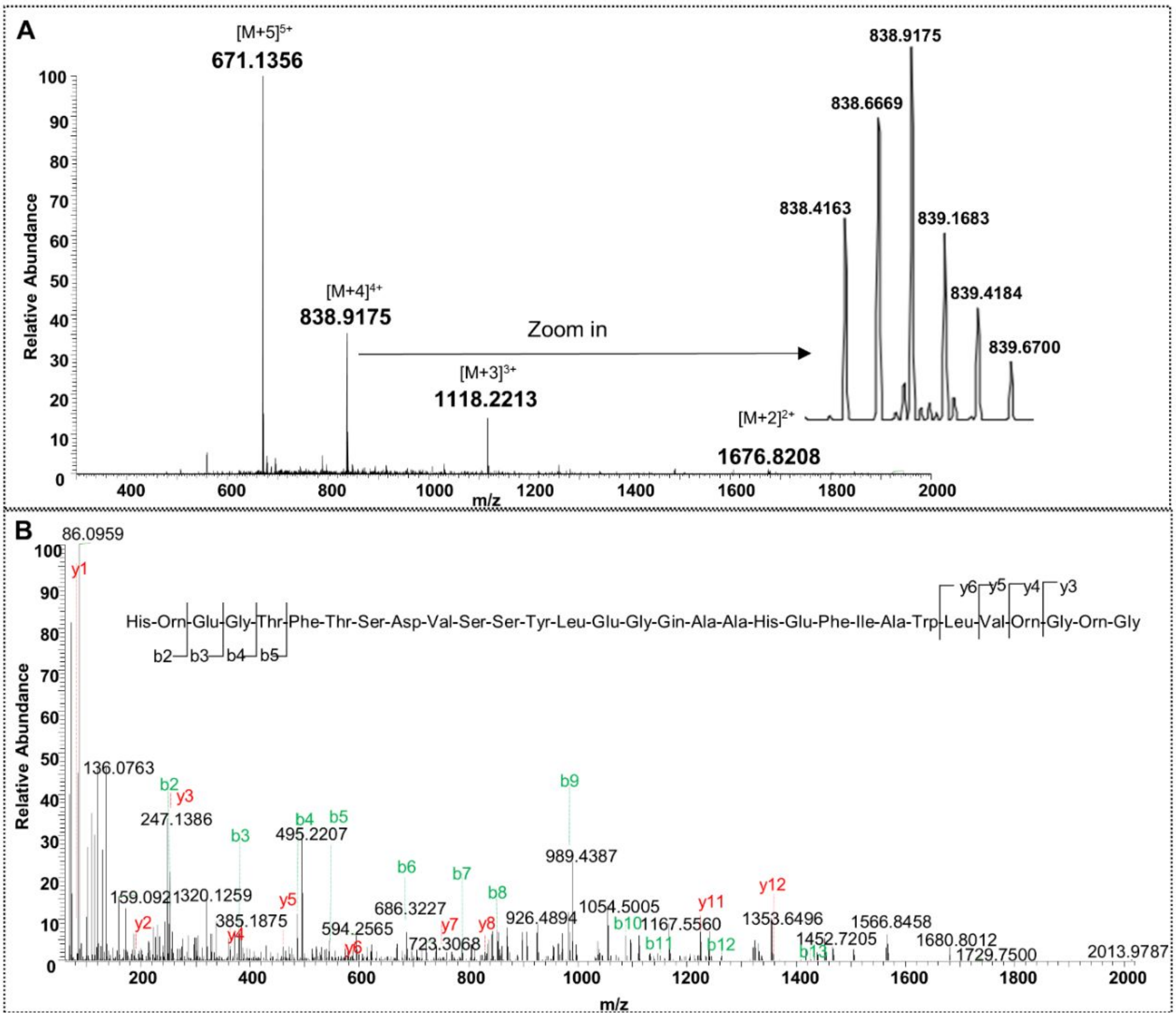

Figure S4. Mass spectrometry of successfully site-specific incorporation of ornithine into core Sem-2R-OspR peptide by pCDF-sem-2R-OspR-mutant.

(A) LC-MS result of Sem-2R-OspR peptide, the peak with a molecular weight of 838.9175 corresponds to the quadruply charged state of the Sem-2R-OspR core peptide, as shown magnified in **Figure S3A**. (B) LC-MS/MS result of Sem-2R-OspR peptide. MS/MS spectrometry analysis revealed fragments with molecular weights of 252.15, 381.19, and 438.21, respectively, indicating the precise insertion of ornithine at position 2 of the core Sem-2R-OspR peptide. This conclusion is further supported by the molecular weights of the b6 and b9 ions, which are clearly visible in the figure. Additionally, the observed y4, y5, and y6 ions, with molecular weights of 361.22, 460.29, and 573.38, respectively, confirm the conversion of the two arginine residues near the C-terminus to ornithine (**Table S4**).

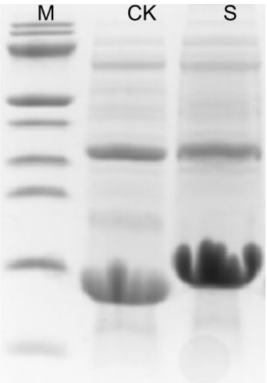

Figure S5 The production of sem-2R-OspR-pAzF precursor peptides with native leader peptide attached in *E. coli* by tricine gels.

The "S" refers to the precursor peptide which purified from the co-transformation of pCDFDuet-26stop-sem-2R-OspR and pEVOL-pAzF plasmids and "CK" indicates the precursor peptide from the transformation of pCDFDuet-26stop-sem-2R-OspR. "M" denotes the molecular weight marker (ladder).
